# Somatic piRNAs and Transposons are Differentially Expressed Coincident with Skeletal Muscle Atrophy and Programmed Cell Death

**DOI:** 10.3389/fgene.2021.775369

**Published:** 2021-12-22

**Authors:** Junko Tsuji, Travis Thomson, Christine Brown, Subhanita Ghosh, William E. Theurkauf, Zhiping Weng, Lawrence M. Schwartz

**Affiliations:** ^1^ Program in Bioinformatics and Integrative Biology, University of Massachusetts Medical School, Worcester, MA, United States; ^2^ Program in Molecular Medicine, University of Massachusetts Medical School, Worcester, MA, United States; ^3^ Department of Neurobiology, University of Massachusetts Medical School, Worcester, MA, United States; ^4^ Department of Biology, University of Massachusetts, Amherst, MA, United States; ^5^ Program in Molecular Medicine, University of Massachusetts Medical School, Worcester, MA, United States

**Keywords:** *Manduca sexta*, development, ping-pong amplification, transposon, RNA interference, small RNAs

## Abstract

PIWI-interacting RNAs (piRNAs) are small single-stranded RNAs that can repress transposon expression *via* epigenetic silencing and transcript degradation. They have been identified predominantly in the ovary and testis, where they serve essential roles in transposon silencing in order to protect the integrity of the genome in the germline. The potential expression of piRNAs in somatic cells has been controversial. In the present study we demonstrate the expression of piRNAs derived from both genic and transposon RNAs in the intersegmental muscles (ISMs) from the tobacco hawkmoth *Manduca sexta*. These piRNAs are abundantly expressed, ∼27 nt long, map antisense to transposons, are oxidation resistant, exhibit a 5’ uridine bias, and amplify *via* the canonical ping-pong pathway. An RNA-seq analysis demonstrated that 19 piRNA pathway genes are expressed in the ISMs and are developmentally regulated. The abundance of piRNAs does not change when the muscles initiate developmentally-regulated atrophy, but are repressed coincident with the commitment of the muscles undergo programmed cell death at the end of metamorphosis. This change in piRNA expression is correlated with the repression of several retrotransposons and the induction of specific DNA transposons. The developmentally-regulated changes in the expression of piRNAs, piRNA pathway genes, and transposons are all regulated by 20-hydroxyecdysone, the steroid hormone that controls the timing of ISM death. Taken together, these data provide compelling evidence for the existence of piRNA in somatic tissues and suggest that they may play roles in developmental processes such as programmed cell death.

## Introduction

Small silencing RNAs are powerful regulators of gene expression. They can lead to epigenetic silencing of transcription, transcript degradation, and inhibition of mRNA translation. The best characterized class of small silencing RNAs are the microRNAs (miRNAs)(Grewal and Elgin 2007; Ghildiyal and Zamore 2009; Castel and Martienssen 2013). miRNAs have been shown to be ∼22 nucleotides (nt) long, ubiquitously expressed and can repress their target transcripts through seed-based partial complementarity (Guo et al., 2010; Ha and Kim 2014; Bartel 2018).

The most recently discovered group of small silencing RNAs are PIWI-interacting RNAs (piRNAs). piRNAs are 23–35 nt in length and predominantly expressed in the germline of animals, including humans (Iwasaki et al., 2015; Williams et al., 2015; Czech and Hannon 2016; Czech et al., 2018). piRNAs guide the PIWI clade of Argonaute proteins to silence transposons and other selfish elements and function to protect the integrity of the germline genome (reviewed in Ozata et al., 2019).

The biogenesis and function of piRNAs has been well studied in the fruit fly *Drosophila melanogaster* (Huang, Fejes Toth et al., 2017). piRNAs are processed from long transcripts that can be up to hundreds of kilobases long that are transcribed from discrete genomic loci termed “piRNA clusters” (Brennecke et al., 2007; Malone et al., 2009; Thomson and Lin 2009). piRNAs are amplified *via* reciprocal target cleavages by PIWI proteins, a mechanism known as the ping-pong cycle (Brennecke et al., 2007; Gunawardane et al., 2007). Because PIWI proteins cleave the phosphodiester bond between the nucleotides in the target RNA that pair with the 10th and the 11th nucleotides of the guide piRNA, and 3′ cleavage products is subsequently made into another piRNA, there is an enrichment of piRNAs that perfectly reverse complement each other in their first 10 nucleotides, the hallmark of the ping-pong cycle. This process typically creates piRNAs with a uridine residue as the first nucleotide of the primary piRNA, and complementarity over the first 10 nt of post-transcriptionally amplified piRNAs (Brennecke et al., 2007; Cora et al., 2014; Wang et al., 2014; Stein et al., 2019).

piRNAs predominantly target transposons, retroviruses and other “selfish” genetic elements. In the absence of piRNAs, transposons can mobilize, resulting in double-stranded DNA breaks in the germline genome leading to infertility (Klattenhoff and Theurkauf 2008; Weick and Miska 2014). Consequently, the expression and function of piRNAs has been most extensively studied in germ cells, gonadal somatic cells, and certain progenitor cells (Brennecke et al., 2007; Rojas-Rios and Simonelig 2018; Sato and Siomi 2020).

While there have been several reports demonstrating the presence of both piRNAs and the associated protein machinery in non-gonadal cells, their role in cellular processes has been a controversial subject (Pandey et al., 2017; Huang et al., 2019; Perera et al., 2019; Onishi et al., 2020; Teefy et al., 2020). Somatic piRNAs have been observed broadly in arthropods, where they may provide genome defense against transposable elements and viruses (Lewis et al., 2018). In agreement with this hypothesis, piRNAs from fat body and midgut cells elicits antiviral response against nucleopolyhedrovirus in the silkmoth *Bombyx mori* (Feng et al., 2020). In addition, piRNAs derived from endogenous viral elements system in the mosquito *Aedes aegypti* help maintain long-lasting adaptive immunity (Whitfield et al., 2017; Tassetto et al., 2019).

In the current study we provide substantial data demonstrating the presence of piRNAs and their synthetic machinery in the intersegmental muscles (ISMs) from the tobacco hawkmoth *Manduca sexta.* We further demonstrate that transposon expression becomes deregulated coincident with the commitment of the muscles to initiate programmed cell death at the end of metamorphosis.

The ISMs are a classical model system for skeletal muscle atrophy and death (Schwartz and Truman 1982; Schwartz 2018; Sheel et al., 2020). These muscles are composed of giant syncytial cells, each of which is about 5 mm long and up to 1 mm in diameter. The ISMs are used by the larvae to crawl and by the developing adult moth to eclose (emerge) from the overlying pupal cuticle at the end of metamorphosis. Three days before adult eclosion (day 15 of the normal 18-day period of pupal-adult development), the ISMs initiate a program of atrophy that results in a ∼40% loss of muscle mass prior to eclosion. This atrophy is non-pathological and the muscles retain normal physiological properties such as resting potential and force per cross-sectional area (Schwartz and Ruff 2002). Late on day 18, coincident with adult eclosion, the ISMs initiate programmed cell death (PCD) and die during the subsequent 30 h. In fact, the term PCD was coined by Lockshin and Williams in 1965 to describe the death of these specific cells (Lockshin and Williams 1965). ISM PCD is a fundamentally different program than atrophy and results in the complete destruction of the contractile apparatus, loss of the resting potential, and enhanced autophagy (Schwartz and Ruff 2002; Tsuji et al., 2020). The dying cells are not phagocytosed and instead activate the molecular machinery required for both cellular destruction and nutrient recycling (Jones and Schwartz 2001; Tsuji et al., 2020). The developmental timing of both atrophy and death are controlled by circadian declines in the circulating titer of the insect molting hormone 20-hydroxyecdysone (20E) (Schwartz and Truman 1983). Judiciously timed administration of exogenous 20E can prevent atrophy or death, and the differential gene expression associated with these programs, but once either of these programs has been initiated, it cannot be altered or delayed by 20E treatment (Tsuji et al., 2020).

Several studies have demonstrated that ISM PCD requires *de novo* gene expression, and numerous death-associated genes have been identified (Schwartz et al., 1990; Schwartz et al., 1990; Tsuji et al., 2020). During the transition from atrophy to death, some ISM transcripts display significant changes in both their stability and translatability that is mediated by their 3′ untranslated regions (UTRs) (Cascone and Schwartz 2001). In subsequent studies, it was shown that specific microRNAs can regulate the translation of death-associated transcripts, establishing a role for this class of small RNAs (Tsuji et al., 2020).

To extend this analysis, we performed RNA-seq with the small RNAs isolated from the ISMs each day of development, ranging from before the initiation of atrophy (day 13) until when the muscles were committed to die (day 18) (Tsuji et al., 2020). We also analyzed ISMs from day 18 animals that had been injected the previous day with 20E to delay cell death. In addition to miRNAs, we also found that the ISMs also contain high levels of piRNAs, an unexpected result. Detailed analysis demonstrated that these piRNAs are: ∼27 nt long, map antisense to transposons, are oxidation resistant, exhibit a uridine bias at their first nucleotide, and amplify *via* the canonical ping-pong pathway. In addition, the ISMs express the genes required for piRNA synthesis and activity. When the ISMs become committed to die, there is a concurrent loss of piRNAs and an apparent deregulation of transposable elements, with repression of some retrotransposons and the induction of DNA transposons. The expression of piRNAs, piRNA regulatory machinery and transposable elements expression are all under the control of 20E. Thus, piRNAs are expressed in somatic tissues where they may regulate developmental processes such as PCD.

## Materials and Methods

### Animals

The tobacco hawkmoth *Manduca sexta* was reared and staged as described previously (Schwartz and Truman 1983). The ISMs are a very discrete cells and can be cleanly isolated without contamination from other tissues. The lateral intersegmental muscles (ISMs) were dissected under ice-cold saline, flash frozen on dry ice, and stored in liquid nitrogen until used for RNA isolation.

Some animals were injected on day 17 of pupal-adult development with 25 µg of 20-hydroxyecdysone (20E) (Sigma) in 10% isopropanol to delay ISM death (Schwartz and Truman 1984) and then the ISMs removed prior to the normal time of eclosion on day 18.

### RNA Isolation, Library Construction and Sequencing

The ISMs of three to four animals per developmental time point (eight time points in total: days 13, 14, 15, 16, 17, 18, and 1-h post-eclosion (PE); plus 20-hydroxyecdysone injection on day 17: 20E) were homogenized and total RNA was isolated using a mirVana RNA Isolation kit (Life Technologies).

For small RNA-seq library construction, 50 µg of total RNA was fractionated by 15% urea polyacrylamide gel electrophoresis and the 18–30 nt fraction extracted for library construction. (While some longer piRNAs may have been missed with this size cut off, it prevented contamination of the library by a ∼32 nt 2S ribosomal RNA that is present in some insect species (Tautz et al., 1988)). 3′ and 5′ adaptors were ligated to the small RNA and the cDNA reverse transcribed and PCR amplified. The libraries were purified by polyacrylamide gel electrophoresis, and subjected to 50 nt single-end sequencing on an Illumina HiSeq™ 2000 (San Diego, CA, United States) by Beijing Genomics Institute (Hong Kong).

For RNA-seq, directional and random primed cDNA libraries were constructed with poly(A)+ RNA, analyzed with a Bioanalyzer (Agilent Technologies; Santa Clara, CA, United States) and 50 nt single-end sequencing was performed as above. For each of the eight time points, we prepared three biological replicates of RNA-seq libraries.

All the sequencing libraries are accessible from Gene Expression Omnibus (GEO) (accession number GSE80830).

### Oxidized Small RNA-Seq Library

piRNAs are 2′-O-methylated at their 3′ termini, which renders them resistant to oxidation (Vagin et al., 2006). In contrast, while oxidation does not destroy miRNAs or mRNA degradation products, it does render them unclonable (Zhang et al., 2014). Therefore, we oxidized small RNAs as outlined in (Gunawardane et al., 2007), then cloned the resulting piRNAs as described above. We sequenced the oxidized small RNA library at the Deep Sequencing Core Facility at the UMass Medical School.

### Genomic Sequence and Annotation Data

We downloaded the genomic assembly of *Manduca sexta* (Msex1.0) and the transcript and the protein sequences (revised-OGS-June2012) from the *Manduca* Base (http://agripestbase.org/manduca/) (Tsuji et al., 2020). The genomic assembly contains 20,868 scaffolds, with a median length of 994 bp. Unfortunately, the annotated *Manduca* genome available lacks the resolution to identify piRNA clusters. We annotated protein-coding genes, transposable elements, low complexity regions, miRNAs, and other non-coding RNAs such as rRNA, tRNA, snoRNA, snRNA etc. The detailed annotation protocol and statistics are described in Supplementary Material (below). All the sequencing libraries are accessible from GSE80830 in Gene Expression Omnibus.

### Sequence Extraction and Annotation of piRNAs

After computationally removing the adaptor sequences, we mapped the extracted sequences to the reference *Manduca* genome using the Bowtie algorithm (Langmead et al., 2009). We only retained reads that matched the genome perfectly for downstream analysis. To identify potential piRNAs, we selected sequences that were longer than 22 nt and not annotated as miRNAs or other non-coding RNAs (*see* Supplementary Material for annotation of miRNAs and other non-coding RNAs). The reads of piRNAs that mapped to multiple loci in the genome were apportioned over these loci, and piRNA reads were normalized by the total number of genome mapping reads excluded other non-coding RNAs (rRNA, tRNA etc.). piRNA abundance is quantified in parts per million (ppm).

### piRNA Ping-Pong Signature

To determine if *Manduca* piRNAs amplify *via* the ping-pong cycle, we computed the Z-score as described in (Li et al., 2009). Briefly, we identified the piRNA pairs that mapped to overlapping genomic positions but on opposite genomic strands. We counted the numbers of such pairs with 5′-5′ overlapping distances from 1 to 20 nts, and calculated Z-score for the 10-nt overlap (the expected overlapping distance due to ping-pong) using the counts of 1–9 nt and 11–19 nt overlaps as the background.

### Relative Mapping Position of piRNAs on Genes

In order to characterize the relative positions in mRNAs that *Manduca* piRNAs map to, we scaled the 5′ UTRs, coding regions, and 3′ UTRs of mRNAs to 350, 1000, and 800 nts respectively, which we calculated are the median lengths of these regions in annotated *M. sexta* genes. Using scaled non-overlapping windows which are equivalent to each of the 2,150 nts of the scaled genes, we counted piRNA abundance in RPKM. For measuring the enrichment, we performed Wilcoxon ranksum test on calculated RPKM values across the 5′ UTRs, coding regions, and 3′ UTRs of mRNAs.

### Gene Expression and Differentially Expressed Genes

We mapped reads in each RNA-seq library to the reference *Manduca* genome using the TopHat2 algorithm (Kim et al., 2013) allowing two mismatches (“-v 2”). To detect reads mapping to transposons, we allowed reads to map to multiple locations of the genome with the “-g” option in TopHat2. The most abundant transposon in *Manduca* is SINE (44,487 copies when all subfamilies are combined) (Supplementary Table S2), we ran TopHat2 with “-g 45,000.” Reads were apportioned by the number of times they mapped to the genome.

After mapping, we counted the number of RNA-seq reads for each gene and transposon, expressed in the unit of RPKM (Reads Per Kilobase of transcript per Million mapped reads). To identify differentially expressed genes and transposons between a pair of time points during ISM development, we ran the DESeq2 algorithm (version 1.5.5) implemented in R (Anders and Huber, 2010) using mapped read counts as the input, taking advantage of the three biological replicates per stage. The statistical analysis was performed by DESeq2 (version 1.5.5). Genes with q-value <0.01 and absolute fold-change (fc) >2 (both computed with DESeq2) were considered to be differentially expressed between the two time points.

## Results

### piRNAs in the Intersegmental Muscles Prior to Atrophy

On day 13 of the pupal-adult development the ISMs are fully functional and have yet to initiate either the atrophy or PCD (Schwartz and Truman 1983). We isolated the 18–30 nt small RNAs at this time point and split the sample into two aliquots. One aliquot was directly cloned and sequenced. The second aliquot was oxidized to render the miRNAs and mRNA degradation products unclonable due to the absence of 2′-O-methylated 3′ termini (Vagin et al., 2006; Grimson et al., 2008). This manipulation preferentially enriches the piRNAs. The small RNA-seq library mapping results are presented in Supplementary Table S1.

**FIGURE 1:**
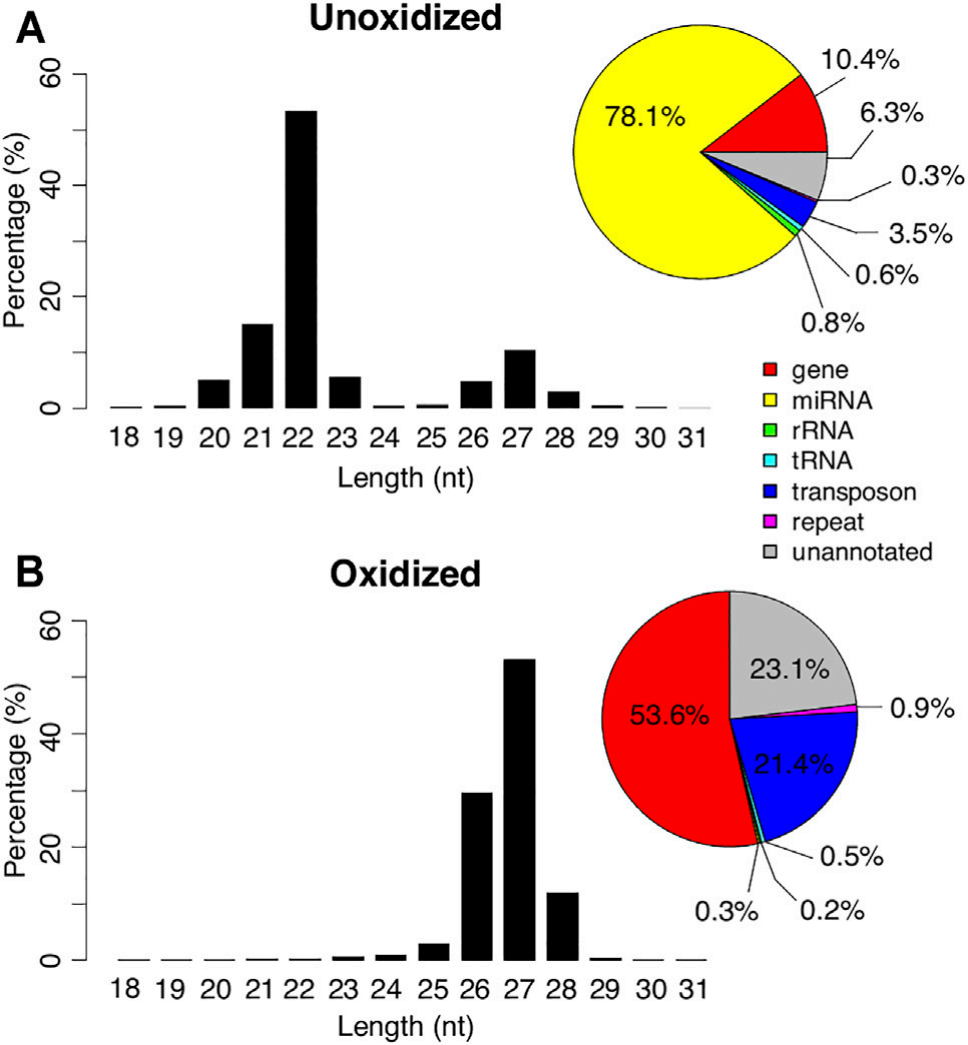
Length and mapping locations of *Manduca* piRNAs. Length distribution of the small RNAs in day 13 unoxidized (top) and oxidized **(bottom)** libraries. Genomic annotations of the locations where the reads mapped are summarized in pie charts **(right)**.

We obtained 17.8 million and 11.4 million reads in the control and oxidized day 13 small RNA libraries respectively, of which 71.7 and 75.8% mapped to the *Manduca* genome. When the mapped reads in the unoxidized small RNA library were partitioned by size, we observed a bimodal distribution, with peaks at 22 and 27 nt (Figure 1). Among the reads shorter than or equal to 23 nt, we annotated 77.7% (78.1% for total reads) as miRNAs (Tsuji et al., 2020). In sharp contrast, among the reads longer than 23 nt, only 0.4% were miRNAs and instead, these RNAs displayed a strong 5′ uridine bias and a weak adenine signal at the 10th position (Supplementary Figure S1).

Oxidation resulted in an almost complete loss of the 22 nt peak and 98.6% of the reads in the oxidized library were >23 nt (Figure 1). The majority of these reads started with a uridine base at the 5′ position (Supplementary Figure S1). Only 0.3% of the reads in this library were identified as miRNAs. In contrast, 53.6% of the reads mapped to genes, 21.4% mapped to transposons, and 23.1% mapped to unannotated regions of the genome (Figure 1). These percentages were much higher than the ≤23 nt reads in the unoxidized library (0.8, 0.3, and 0.6% respectively), and comparable to the reads >23 nt in the unoxidized library (9.7, 3.2, and 5.6% respectively). Based on size and resistance to oxidation the 27 nt peak appears to represent piRNAs.

### piRNA Expression Changes During the ISM Development

In addition to the libraries on day 13, we generated and sequenced unoxidized small RNA libraries from seven more time points of ISM development: days 14, 15, 16, 17, 18, 1-h post-eclosion (PE) and 20-hydroxyecdysone (20E) treated animals. Similar to what we observed with the day 13 unoxidized sample, the small RNAs from the other time points also displayed a bimodal distribution of small RNAs, with the majority of the ∼22 nt sequences mapping to miRNAs (Supplementary Figure S1). In all of these samples, there was a strong bias for uridine in the first nucleotide, especially for the piRNAs (reads >23 nt).

**FIGURE 2:**
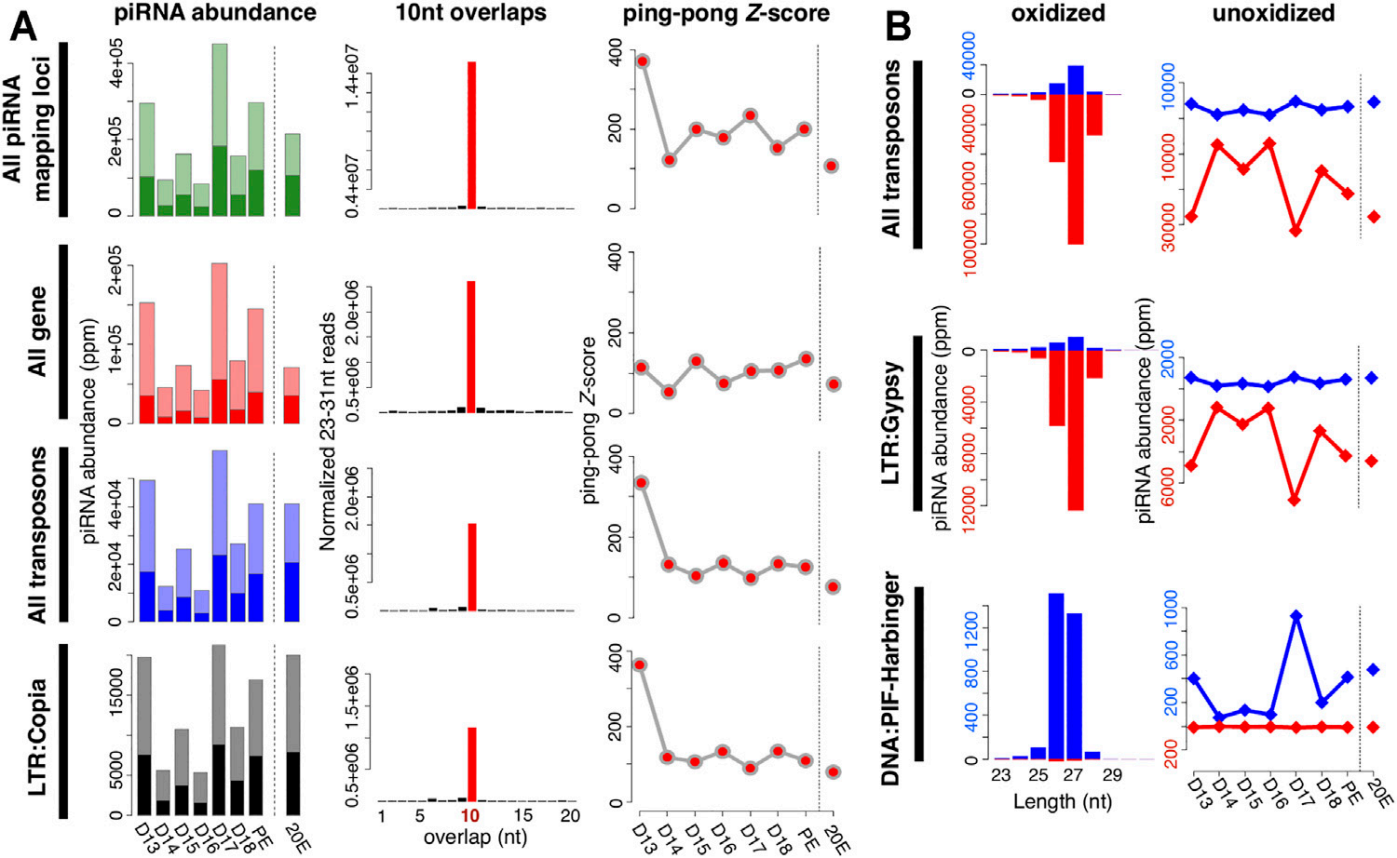
Characteristics of piRNAs expressed in the ISM. **(A)** Changes in piRNA abundance during the ISM development **(left)**; the frequency of 5′-5′ 10 nt overlaps between piRNA pairs **(middle)**; and the corresponding ping-pong Z-scores during development **(right)**. The shaded bars on the left panels indicate the fraction of piRNAs that are amplified *via* the ping-pong amplification loop. Almost all of the Z-scores were statistically significant (red dots). **(B)** Size distribution and strand bias of piRNAs mapped to transposons **(left)**, and the change in piRNAs expression during the ISM development **(right)**. Blue reflects sense strand bias while red is indicative of antisense. As examples, piRNAs mapped to LTR/Gypsy are in the upper panel while those mapping to DNA/PIF-Harbinger are on the bottom panel.

piRNAs abundance from each stage was normalized by the sequencing depth and calculated as “parts per million” (ppm). The expression of both genic and transposable element piRNAs gradually declined from day 13 to day 16, increased sharply on day 17, and then declined dramatically on day 18 (Figure 2A). piRNA abundance was elevated in the 1-h post-eclosion (PE) sample. While treatment with 20E on day 17 delays ISM death (Schwartz and Truman 1983), it did not significantly alter piRNA abundance in the muscles relative to the corresponding PE timepoint. Interestingly the number of genic piRNAs was greater than those that map to transposons, the significance of which is unknown (Figure 2A).

Of the 64 known transposon families in *Manduca*, 34 generated piRNAs that mapped to the genome. Among them, piRNAs for 27 transposon families mirrored the pattern of expression observed for all piRNAs and transposon-mapping piRNAs (e.g. LTR:Copia in Figure 2A) during ISM development. In contrast, the expressions of piRNAs mapped to five transposon families (two DNA transposon families: P and TcMar; and two LINE families: CR1 and I; and one SINE family: tRNA) were reduced gradually during the ISM development (Supplementary Figure S2).

### piRNAs Predominantly Map Antisense to Transposons

The majority of piRNAs in flies that map to transposons are antisense to the mRNAs in order to guide the PIWI-family proteins Aub and PIWI to repress transposon expression (Czech and Hannon 2016). Consequently, mutations in piRNA pathway proteins that alter this antisense bias, such as Qin, lead to transposon de-repression (Zhang et al., 2011). We examined the strand bias of transposon-mapping piRNAs at each stage of ISM development. Of the 34 transposon families with mapped piRNAs, we found that the majority of them (28 families) displayed strong antisense bias, while the remaining six families displayed a sense bias. The antisense piRNAs to transposons tend to be 27 nt in length (e.g. LTR:Gypsy in Figure 2B), and the sense piRNAs to transposons tend to be either 26 or 27 nt (e.g. DNA:PIF-Harbinger in Figure 2B). Although the relative abundance of piRNAs was regulated during the course of ISM development their length distributions were constant.

### piRNAs Expressed in the ISM Amplify *via* the Ping-Pong Amplification Loop

Following transcription from piRNA clusters, the primary transcripts are processed and cyclically amplified by a mechanism known as the “ping-pong amplification loop” (Meister 2013; Czech and Hannon 2016). The secondary piRNAs generated *via* ping-pong tend to have a 10 nt 5′ end overlap with other piRNAs on the opposite strand. This ping-pong activity is quantified by calculating the frequency of 5′-5′ overlaps between piRNA pairs, normalized as a Z-score (Li et al., 2009).

We detected high Z-scores at all stages examined. For example, piRNAs from day 17 had an overall Z-score of 234.6 (Figure 2A). piRNAs from both transposons and genic sequences were amplified by ping-pong as demonstrated by Z-scores of 98.7 and 105.5 respectively. Z-scores were high on day 13, fell precipitously during the next 3 days, and then rose on day 17 and remained high for the rest of development, which agrees well with the abundance of piRNAs in the tissue. It should be noted that these developmental changes in Z-score were not artifacts arising from variations in sequencing depth since these same patterns were observed when we down-sampled to 8 million reads per stage prior to our analysis.

As part of our analysis, we computed the Z-scores for piRNAs that mapped to each transposon class. Out of 34 transposon families with detectable piRNAs, 20 families displayed a high ping-pong signature throughout ISM development (e.g. LTR:Copia in Figure 2A), and 14 families displayed statistically significant Z-scores at least transiently. Intriguingly, in 12 out of 14 transposon families, the ping-pong signature peaked on day 17 in advance of the commitment of the ISMs to die. These data support the hypothesis that ISM piRNAs amplify *via* the traditional ping-pong amplification loop.

### Genic piRNAs Preferentially Map to 5′UTRs

Combining the RepeatMasker result with our own gene annotation, 8,633 (45.9%) of the 18,806 genes in *M. sexta* contain transposons within their introns. From the mapping results with our oxidized small RNA-seq library, we observed that piRNAs (24.38 ppm per intron in median) fell into the transposon-derived introns from 6,902 genes (80.0% of 8,633 genes containing transposons). piRNAs also mapped to introns that did not contain transposons, although the abundance was far lower (1,731 out of 18,806 genes (9.2%)). Compared to the abundance of piRNA reads on transposon-derived introns, there were very few non-transposon introns (2.29 × 10^−2^ ppm per intron in median).

It has been demonstrated that genic piRNAs tend to map to the sense orientation of 3′UTRs in germline and somatic cells (Robine et al., 2009; Jones et al., 2016; Sokolova et al., 2019). However, in the ISMs, we observed that the genic piRNAs tended to map to the sense strand preferentially within the 5′UTRs rather than the 3′ UTRs or coding sequences (CDSs). The strand bias patterns of the genic piRNAs uniquely mapped to 5′UTRs, CDSs, 3′UTRs, and introns were the same as those with all genic piRNA reads. We then focused specifically on the top 25% of the genes that are highly enriched with piRNAs. In the oxidized small RNA-seq library, 5′UTRs tended to be more enriched with piRNAs mapped to the sense strands of genes as compared to other gene domains. In unoxidized small RNA-seq libraries, the overall trends were stably observed through all time points across development.

**FIGURE 3:**
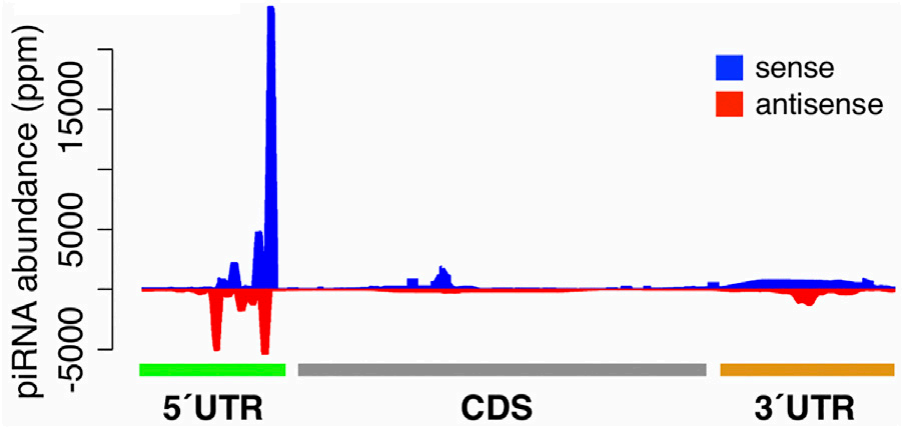
Relative mapping positions of genic piRNAs. The relative mapping position of piRNAs on highly expressed genes. piRNAs mapping to the sense and antisense strands of genes are highlighted in blue and red respectively.

Considering the result that genic piRNAs tend to fall into the sense strands of 5′UTRs, we investigated the relative mapping positions of piRNAs in genes by examining the oxidized small RNA-seq library from day 13. Interestingly, when the piRNAs that mapped to highly expressed genes were plotted onto the gene map, there was a striking peak in the sense orientation in the 5′UTRs (Figure 3).

(Only a modest number of piRNAs mapped to low abundance transcripts). In unoxidized small RNA-seq libraries, genic piRNAs tended to map to 5′UTRs and to form the peaks of mapped piRNAs (Supplementary Figure S3).

We also sought to determine if genic piRNA expression was correlated with the abundance of their source mRNAs. Toward that end, we examined the top 25% piRNA-generating genes that were either up- or down-regulated between days 17 and 18. (It should be noted that none of the piRNA pathway related genes were on the list, suggesting that genic piRNAs are unlikely to alter the abundance of the piRNA pathway machinery). As shown in Supplementary Figure S4, piRNA generation levels were unrelated to the RKPM levels. The RPKM distribution of the top 25% piRNA generating mRNAs ranged from 0.089 to 13,773.77 RPKM (median = 15.45 RPKM).

### piRNA Biogenesis Pathway Factors Are Differentially Expressed During ISM Atrophy and the Commitment to Die

We next sought to determine which piRNA pathway protein components are expressed in the ISMs. Based on piRNA pathway genes characterized in *Drosophila*, we identified all 19 of the genes we sought in the ISMs (*ago3*, *armi*, *aub*, *piwi*, *BoYb*, *egg*, *krimp*, *mael*, *papi*, *qin*, *rhi*, *shu*, *spn-E*, *tej*, *tud*, *vas*, *vret, hen-1*, and *zuc*), plus two small RNA biogenesis pathway factors (*ago1* and *ago2*). In agreement with published reports, we only identified a single Aub/PIWI protein sequence, which is also the case for other Lepidopterans like *Tricoplusia ni* and *Bombyx mori* (Cao et al., 2015). Consequently, we refer to this protein as Aub/PIWI.

**FIGURE 4:**
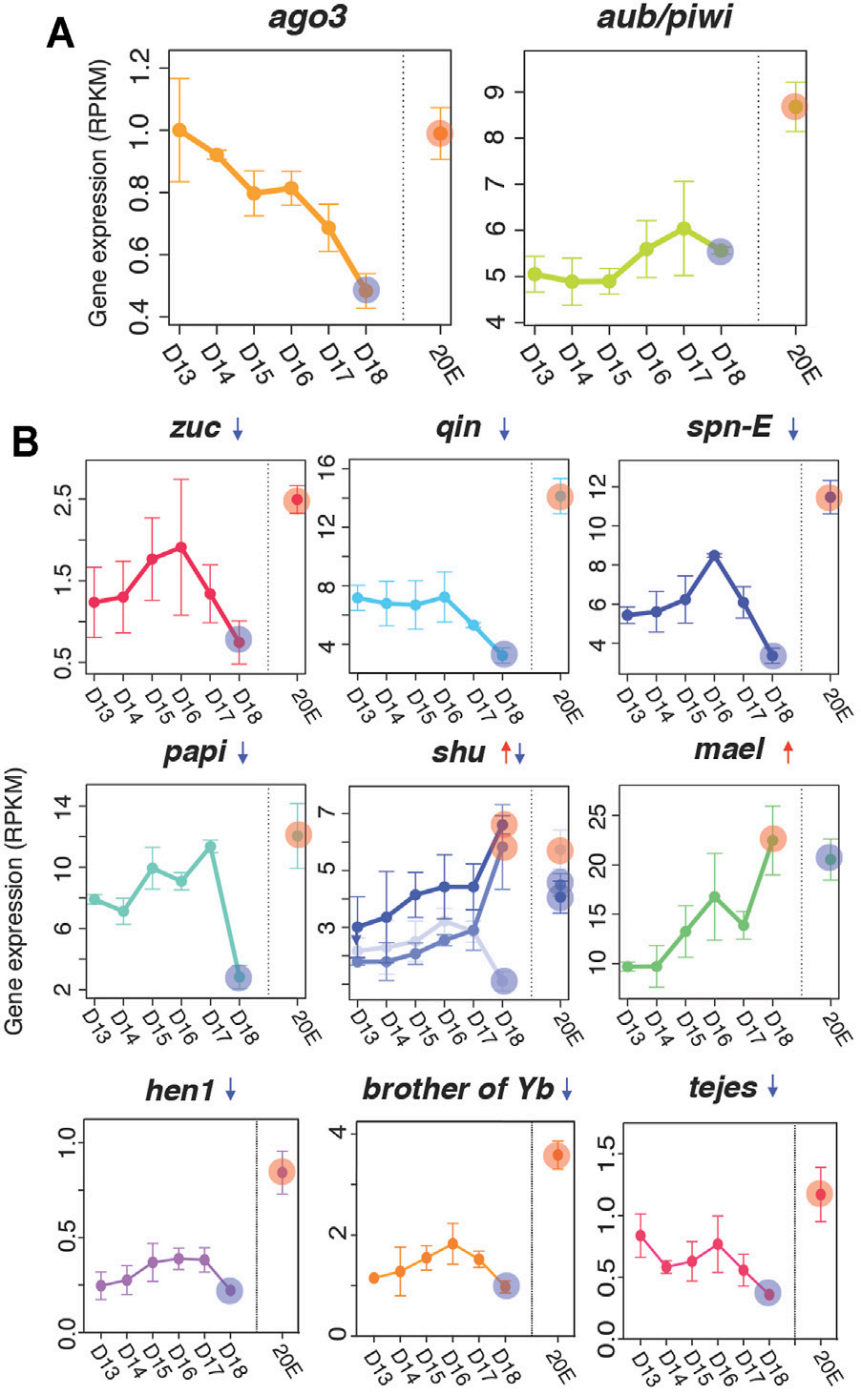
Expression of piRNA biogenesis pathway factors. **(A)** Gene expression of *ago3* and *aub/piwi* based on RNA-seq analyses **(top)** **(B)** Downregulated (*zuc*, *qin*, *spn-E*, *hen1*, *brother of Yb*, *tejes* and *papi*), mixed (*shu*), and upregulated (*mael*) piRNA pathway factors in the ISMs prior to the initiation of cell death. The difference between each developmental time point (days 13–17) were statistically different from the day 18 value, although we only highlighted the comparison of day 13–day 18 (red and blue circles). Red and blue circles on day 18 indicate up- and down-regulation of the factors compared to expression on day 13. Those circles in the 20E lane indicate statistically significant changes in ISM gene expression animals injected on day 17 with 20E to delay cell death on day 18. Means are presented plus/minus standard deviations calculated from three replicates of the mRNA-seq libraries.

Next, we examined the mRNA-seq reads from six developmental stages (days 13, 14, 15, 16, 17, 18) plus 20E-treated to determine if these factors are differentially expressed. While the expression levels of *ago3* and *aub* appear to be low, their expression was nevertheless ranked within the top 64.4 and 31.1% respectively of all expressed genes (Figure 4A). The expression levels of other genes in this pathway were also within the top 18–65% of all expressed genes (Supplementary Figure S4).

Computational analysis suggests that the expression of many of the small RNA biogenesis pathway factors were developmentally regulated, with a general trend for stable or increasing expression prior to the initiation of atrophy (days 13–15), a sharp decline on day 17, and a near loss of expression when the ISMs became commitment to die on day 18. Four genes were significantly repressed on day 18: *papi* (*q* = 5.8 × 10^−9^, fc = −2.6), *qin* (q = 2.9 × 10^−7^, fc = −2.1), *zuc* (q = 3.9 × 10^−3^, fc = −2.1), and *spn-E* (q = 1.7 × 10^−9^, fc = −2.0) (Figure 4B) and five other demonstrated this general trend (*ago3*, *armi*, *egg*, *krimp*, and *tud*), although their changes did not reach statistical significance (Supplementary Figure S5). In contrast, *mael* mRNA levels were increased with the commitment to die (q = 6.4 × 10^−19^, fc = 2.5; Figure 4B) as were some members of the *shu* family (Figure 4B). In all cases, expression of piRNA pathway components were regulated by 20E and displayed their highest levels of expression when exposed to the exogenous steroid, suggesting that these genes are regulated by the same developmental signals that control developmental changes in the ISM, including atrophy and death.

### Transposon Expression Becomes Deregulated when the ISM Become Committed to Die

**FIGURE 5:**
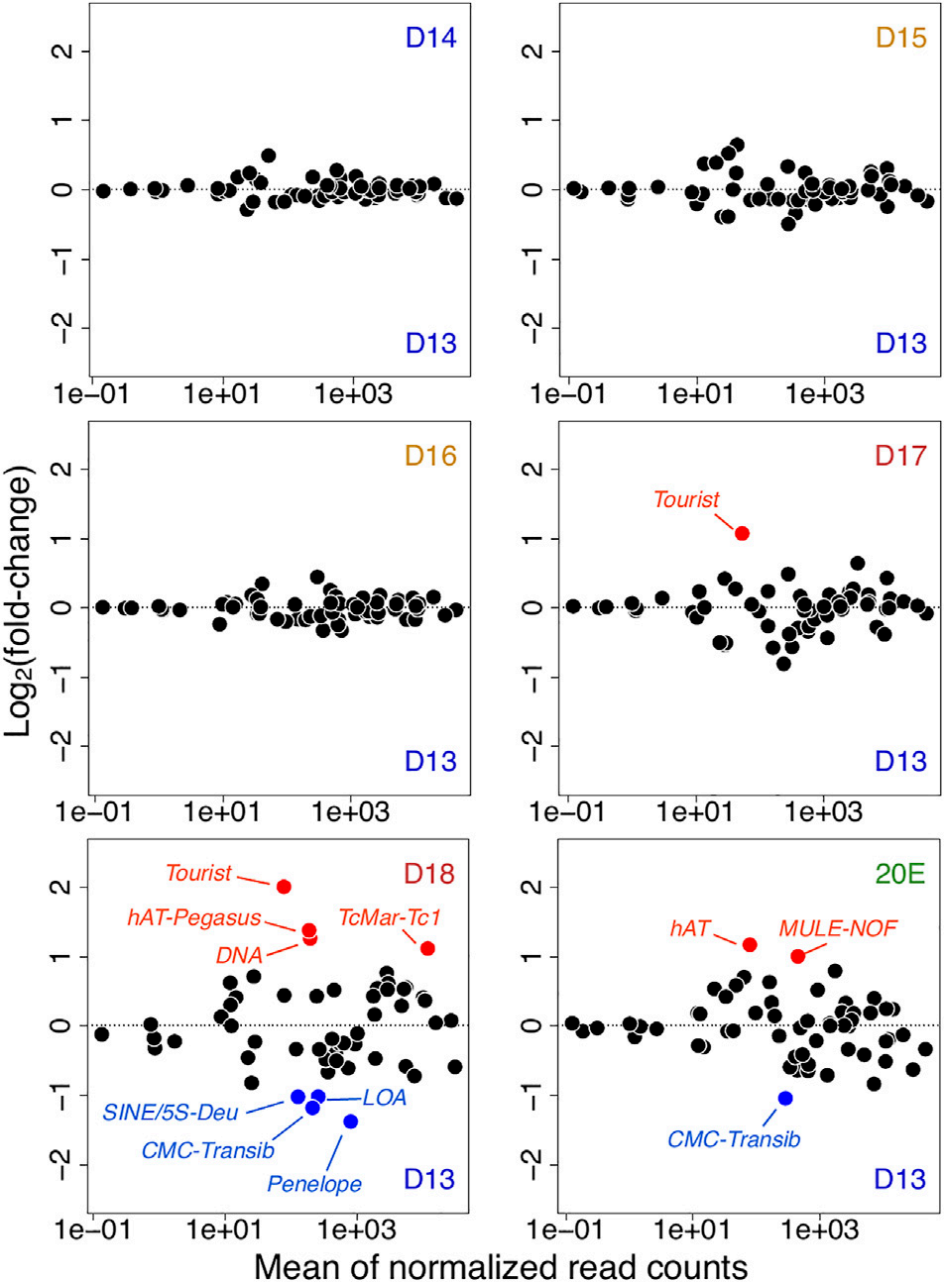
Transposon expression in atrophy and ping-pong piRNAs. Changes in transposon expression during ISM development relative to day 13 (from day 14 to day 18, and 20E). Up-regulated and down-regulated transposons are highlighted in red and blue respectively.

We observed that the abundance of both piRNAs, and the majority of the factors that mediate their biogenesis, declined precipitously coincident with the commitment of the ISMs to die on day 18. Consequently, we sought to test the hypothesis that this loss might be correlated with changes in transposon expression. Using the RNA-seq reads mapped to transposon loci as the input, we computed the expression of each transposable element and the fold change during ISM development relative to day 13 (from day 14 to day 18, and 20E) (Figure 5).

Prior to day 18, the only significant change was the Tourist DNA mobile element, which was up-regulated on day 17 (q = 7.0 × 10^−7^, fc = 2.1) compared to day 13. Coincident with the muscle’s commitment to die on day 18, (but prior to the initiation of death later in the day), the patterns of the transposon expression changed dramatically. Four DNA transposable elements were up-regulated compared to those on day 13: Tourist (*q* = 8.0 × 10^−13^, fc = 4.0), hAT-Pegasus (*q* = 4.1 × 10^−5^, fc = 2.6), TcMar-Tc1 (*q* = 6.4 × 10^−12^, fc = 2.2), and general DNA mobile elements (*q* = 4.6 × 10^−11^, fc = 2.4). Concurrently, the expression levels of four transposons were significantly down-regulated on day 18 as compared to day 13: 5S-Deu (SINE; *q* = 4.7 × 10^−4^, fc = −2.0), LOA (LINE; *q* = 1.1 × 10^−8^, fc = −2.0), Penelope (LINE; *q* = 3.1 × 10^−6^, fc = −2.6), and CMC-Transib (DNA transposon; *q* = 4.7 × 10^−4^, fc = −2.3). Interestingly, pre-treatment with the steroid 20E, which delays ISM death, muted differential transposon expression of transposons.

## Discussion

In this report we present substantial evidence supporting the presence of developmentally-regulated piRNAs in striated skeletal muscle, a highly differentiated somatic cell. Like piRNAs characterized from ovary and testis, *Manduca* ISM piRNAs: are ∼27 nucleotides long, have a strong 5’ uridine bias, are oxidation resistant, amplify *via* a ping-pong mechanism, map to transposable elements, and are predominantly antisense. (We were unable to determine if these piRNAs physically associated with Aub as none of the anti-*Drosophila* Aub antibodies that we tested recognized *Manduca* Aub/PIWI (data not shown)). While piRNAs from *Manduca* ovary have not been examined, ovarian piRNAs in the related Lepidopteran *Bombyx mori* are also ∼27 nt long, map antisense to transposons, and amplify *via* ping-pong mechanism (Kawaoka et al., 2011).

ISM piRNAs represent a substantial percentage of the small RNA pool within the tissue and are neither low abundance sequences nor potential contaminants from mRNA degradation, as oxidized mRNA degradation products cannot be cloned (Zhang et al., 2014). The ISMs are a very large and discrete tissue that can be isolated cleanly from the animal at each stage of development without contamination from other piRNA-rich tissues such as gonads (Schwartz 2018). (Indeed, gonad-specific transcripts, such as the vitellogenin receptor and caudal, which are restricted to the ovary, are expressed at 0.01–0.04 RPKM during the 6 days of development examined in our study, which are orders of magnitude lower than the majority of the piRNA pathway genes that we analyzed). As well, unlike mammalian muscle, the ISMs are composed of only a single fiber type and do not contain regenerative stem cells like satellite cells, endothelial cells or pericytes that might complicate the analysis (Beaulaton and Lockshin 1977).

The expression of piRNAs and the machinery required for their synthesis and action in somatic cells is controversial and the subject of debate (Pandey et al., 2017; Lewis et al., 2018; Onishi et al., 2020). Some studies have suggested that piRNAs may act in the nervous system and influence transposon activity as well as aspects of learning and memory. For example, in *Drosophila*, the piRNA pathway proteins Aub and Ago3 regulate transposon expression and mobilization in the mushroom bodies, brain structures that regulate memory formation and cognitive function (Perrat et al., 2013). These proteins are also found in specialized structures within glial cells in the adult *Drosophila* brain where they appear to repress transposon activity (Tindell et al., 2020). In the sea slug *Aplysia*, a specific piRNA has been shown to modulate synaptic plasticity and memory (Lee et al., 2011; Rajasethupathy et al., 2012).

piRNAs have been also reported in cancer cells (Meister, 2013) and regenerative stem-like cells in invertebrates (Kim et al., 2020). For example, PIWI-like proteins and piRNAs have been identified in somatic cells of the Cnidarian *Hydra* and the flatworm *Macrostomum*, although it is thought that their expression is restricted to stem-like progenitor cells that have the capacity to regenerate all cell types of the animal, including gonad (Juliano et al., 2014; Zhou et al., 2015). Loss of somatic piRNA pathway in *Drosophila* fat body disrupts metabolic homeostasis by depleting lipids and stored metabolites leading shortened lifespan (Jones et al., 2016). In the silkmoth *Bombyx mori*, the primary sex-determining factor is regulated by a single piRNA originated from the sex-determining genomic locus of W chromosome (Kiuchi et al., 2014). In the nematode *C. elegans*, the piRNA pathway components, including PRDE-1 and PRG-1, are expressed in neurons and their inhibition facilitates sensory neuron regeneration following injury in a cell autonomous manner (Kim et al., 2018).

The best characterized system for the analysis of somatic piRNAs is follicle cells in the *Drosophila* ovary (Olivieri et al., 2010; Ishizu et al., 2015; Onishi et al., 2020). Follicle cells are epithelial cells that are needed for the survival and maturation of the underlying germline cells, and disruption of the piRNA pathway in these cells leads to enhanced transposon activity and sterility (Olivieri et al., 2010). While follicle cells are derived somatically, they are physically connected to the germline *via* large ring canals, so it is not clear how much interplay may exist between them. Nevertheless, piRNA production can take place in the follicle-derived OSS (ovary somatic sheet) cell line, supporting the hypothesis that these specialized somatic cells can produce piRNAs (Pelisson et al., 2007; Li et al., 2009; Saito et al., 2009; Sato and Siomi 2020). However, piRNA production in these cells differs from that seen in ovarian cells in several key ways (Li et al., 2009; Malone et al., 2009). First, follicle cells lack both Aub and Ago3, and consequently do not display ping-pong amplification (Malone et al., 2009). Secondly, these piRNAs are expressed predominantly from uni-strand genomic piRNA clusters, while ovarian cells produce piRNAs from both single and double-stranded transposable element clusters (Malone et al., 2009; Yashiro et al., 2018). In contrast to follicle cells, the ISMs express both *Aub* and *Ago3* and display efficient ping-pong amplification with high Z-scores. These data suggest that while the ISMs are clearly somatic cells, their piRNA pathway more closely represents the hallmarks of germline piRNA production.

Sequencing and mapping analyses have demonstrated that *Manduca* piRNAs are generated from both transposable elements and protein coding genes. In the few instances where genic piRNAs have been analyzed, they have been shown to be derived predominantly from the 3′ UTR sequences within mRNAs (Robine et al., 2009; Saito et al., 2009; Jones et al., 2016; Sokolova et al., 2019). In contrast, genic piRNAs analyzed from *Manduca* appear to be derived primarily from the start of translation in the 5′UTR. The possible regulatory role that these sequences might serve is unclear.

The ISMs are a classic model system for the study of skeletal muscle atrophy and death (Finlayson 1956; Lockshin and Williams 1965; Schwartz et al., 2016). Data presented here demonstrate that the expression of ISM piRNAs is developmentally-regulated, with low levels during the window when the ISMs undergo developmentally-regulated atrophy and peak expression on day 17, prior to the time when the ISMs become committed to die late on day 18. Consequently, we examined the expression of transposable elements since they are the primary target of piRNAs. Transposon expression appears to be tightly controlled in the ISMs since there was almost no variation their abundance until day 17, at which point they became deregulated. Within a matter of hours, the expression of several DNA transposons increased significantly, while some elements, most notably retrotransposons, were concurrently repressed. The reason for this disparity is not known, but DNA elements integrate directly into the genome *via* a cut-and-paste mechanism, while retrotransposons may integrate *via* a copy-and-paste mechanism (Sultana et al., 2017). The ability of the ISMs to initiate PCD occurs when the circulating levels of the steroid hormone 20E decline below a specific threshold on day 17 (Schwartz and Truman 1983). Hormone replacement with exogenous 20E on day 17 not only delays ISM death, it also prevented the developmental changes in the expression of piRNAs, piRNA protein machinery and transposon. This data supports the hypothesis that the piRNA pathway is also under hormonal control. It should be noted that the changes in transposon expression occur well in advance of the initiation of cell death and therefore do not appear to be a secondary consequence of cellular suicide. In the germline, transposon expression is repressed in order to protect the genome from insertional mutagenesis and subsequent catastrophe. However, this same process might confer an advantage for the organism by ensuring that cell death is indeed an irreversible process. For example, during apoptosis, genome destruction is insured by the activation of endogenous nucleases that cleave chromosomal DNA into nucleosome sized fragments (Wyllie et al., 1980). However, the ISMs die by an autophagic process that does not include DNA fragmentation (Beaulaton and Lockshin 1977; Schwartz et al., 1993; Schwartz et al., 2016). One intriguing hypothesis is that transposon expression and reintegration might serve a similar role for cells undergoing non-apoptotic forms of cell death where it insures that condemned cells are truly “dead” by fragmenting the genome and depleting the cell of beta-nicotinamide adenine dinucleotide and ATP (Berger 1985). Indeed, some support for this hypothesis comes from the observation that transposon expression is elevated in certain neurodegenerative disorders (Li et al., 2012; Tan et al., 2012; Jonsson et al., 2020), a phenomenon that has been called a “transposon storm” (Reilly et al., 2013; Dubnau 2018). To date, none of these studies have examined the possible expression of piRNAs in the diseased tissues. We attempted to directly test the hypothesis that reintegration of transposons in *Manduca* would result in double stranded breaks in genomic DNA by sequencing genomic libraries generated from ISMs taken before and after adult eclosion. Unfortunately, laboratory reared *Manduca* are not isogenic, and the individual-to-individual variability in transposon copy number precluded a direct test of the hypothesis (data not shown). As well, we sought to use antibodies against phosphorylated histone H2Av since it is a marker of double stranded DNA breaks (Madigan et al., 2002), but the antibodies directed against *Drosophila* phosphorylated H2Av did not cross react with the *Manduca* protein, and a Lepidopteran-specific antibody has not been identified (data not shown). In the absence of data demonstrating that the elevated expression of transposons is associated with enhanced genomic integration, we can only speculate about their possible role(s) in the cell death process.

Taken together, we have presented compelling evidence that piRNAs are expressed in highly differentiated somatic cells. Their expression is developmentally regulated and controlled by the steroid hormone 20E. piRNA expression is repressed coincident with the commitment of the ISMs to die, which is correlated with the deregulation of transposon expression. These data verifies the expression of somatic piRNAs and suggest the intriguing possibility that they impact the process of cell death during development, and perhaps pathogenesis.
