# Supplementary material for: Somatic piRNAs and Transposons are Differentially Expressed Coincident with Skeletal Muscle Atrophy and Programmed Cell Death
Source: Front Genet. 2021 Dec 22;12:775369. doi: 10.3389/fgene.2021.775369 (PMC8730325; doi:10.3389/fgene.2021.775369)
Supplement: Supplementary file 1 [file Table1.DOCX]

| **ISM stage** | **Sequenced** | **Mapped** | **ncRNA matching reads** | **Small RNA reads (excluding ncRNAs)** | **miRNA matching reads** | **Reads excluding ncRNAs and miRNAs** | **Transposon matching reads** | | | **Gene matching reads** | | |
| --- | --- | --- | --- | --- | --- | --- | --- | --- | --- | --- | --- | --- |
|  |  |  |  |  |  |  | **Total** | **Sense** | **Antisense** | **Total** | **Sense** | **Antisense** |
| Day 13  (ox) | 11,429,267 | 8,668,354 | 61,001 | 8,607,353 | 37,081 | 8,570,272 | 1,929,542 | 437,865  (372,365) | 1,622,677  (1,557,177) | 4,575,106 | 2605343  (1,697,683) | 3,785,083  (2,877,423) |
| Day 13 | 17,804,374 | 12,756,711 | 191,882 | 12,564,829 | 9,965,310 | 2,599,519 | 434,374 | 78,575  (66,217) | 380,515  (368,157) | 1,245,475 | 692846  (447,322) | 1,043,677  (798,153) |
| Day 14 | 17,754,434 | 11,535,537 | 207,846 | 11,327,691 | 10,297,045 | 1,030,646 | 104,714 | 18,778  (15,482.5) | 92,527  (89,231.5) | 345,005 | 20879  (134,894) | 284,011  (210,112) |
| Day 15 | 16,017,264 | 10,496,112 | 238,046 | 10,258,066 | 8,970,081 | 1,287,985 | 184,436 | 32,733  (28,641.5) | 159,886  (155,794) | 522,516 | 283438  (181,974) | 442,007  (340,542) |
| Day 16 | 15,486,380 | 10,588,280 | 132,532 | 10,455,748 | 9,611,328 | 844,420 | 90,070 | 14,863  (12,599) | 79,735  (77,471) | 300,770 | 181427  (117,292) | 247,614  (183,478) |
| Day 17 | 21,352,266 | 15,004,690 | 201,824 | 14,802,866 | 10,520,008 | 4,282,858 | 576,891 | 95,373  (85,154.5) | 501,955  (491,736) | 1,932,158 | 1,021,475  (662,226) | 1,629,182  (1,269,930) |
| Day 18 | 25,223,522 | 20,267,775 | 217,557 | 20,050,218 | 17,458,186 | 2,592,032 | 369,924 | 69,297  (60,350.5) | 318,520  (309,574) | 1,058,573 | 592,770  (380,844) | 889,654  (677,728) |
| PE | 21,384,271 | 17,190,147 | 226,160 | 16,963,987 | 13,556,249 | 3,407,738 | 446,930 | 77,826  (67,861) | 389,034  (379,069) | 1,493,902 | 828,185  (533884) | 1,254,318  (960,018) |
| 20E | 24,107,589 | 19,446,305 | 260,663 | 19,185,642 | 15,388,282 | 3,797,360 | 666,492 | 130,861  (112,564) | 572,226  (553,928) | 1,782,711 | 965,013  (620,772) | 1,506,179  (1,161,940) |
