# Supplementary material for: Somatic piRNAs and Transposons are Differentially Expressed Coincident with Skeletal Muscle Atrophy and Programmed Cell Death
Source: Front Genet. 2021 Dec 22;12:775369. doi: 10.3389/fgene.2021.775369 (PMC8730325; doi:10.3389/fgene.2021.775369)
Supplement: Supplementary file 2 [file Table2.DOCX]

| **Transposon families** | **Copy number** | **Total bases (bp)** | **Fraction in the genome (%)** |
| --- | --- | --- | --- |
| DNA / Academ | 267 | 60,804 | 1.4497e-2 |
| DNA / Chapaev-Chap3 | 4 | 1,593 | 3.7981e-4 |
| DNA / CMC-Chapaev | 67 | 4,466 | 1.0648e-4 |
| DNA / CMC-Chapaev-3 | 160 | 43,665 | 1.0411e-2 |
| DNA / CMC-EnSpm | 11 | 778 | 1.8549e-4 |
| DNA / CMC-Transib | 78 | 22,779 | 5.4310e-3 |
| DNA / Crypton | 1 | 44 | 1.0491e-5 |
| DNA / hAT | 2 | 124 | 2.9564e-5 |
| DNA / hAT-Ac | 120 | 15,125 | 3.6061e-3 |
| DNA / hAT-Blackjack | 2 | 156 | 3.7194e-5 |
| DNA / hAT-Charlie | 1,590 | 205,679 | 4.9038e-2 |
| DNA / hAT-hATm | 108 | 7,234 | 1.7248e-2 |
| DNA / hAT-hobo | 8 | 445 | 1.0610e-4 |
| DNA / hAT-Pegasus | 61 | 5,750 | 1.3709e-3 |
| DNA / hAT-Tip100 | 1 | 70 | 1.6690e-5 |
| DNA / Kolobok-Hydra | 32 | 1,996 | 4.7589e-4 |
| DNA / Maverick | 120 | 16,207 | 3.8641e-3 |
| DNA / Merlin | 1 | 82 | 1.9551e-5 |
| DNA / MULE-NOF | 20 | 1,385 | 3.3021e-4 |
| DNA / P | 34 | 2,106 | 5.0212e-4 |
| DNA / PIF-Harbinger | 59 | 7,868 | 1.8759e-3 |
| DNA / PIF-ISL2EU | 23 | 5,662 | 1.3500e-3 |
| DNA / PiggyBac | 53 | 14,777 | 3.5232e-3 |
| DNA / Sola | 710 | 70,726 | 1.6863e-2 |
| DNA / TcMar | 112 | 11,173 | 2.6639e-3 |
| DNA / TcMar-Fot1 | 28 | 1,972 | 4.7017e-4 |
| DNA / TcMar-Mariner | 1,953 | 332,349 | 7.9239e-2 |
| DNA / TcMar-Pogo | 1 | 53 | 1.2636e-5 |
| DNA / TcMar-Tc1 | 1,234 | 424,500 | 0.10121 |
| DNA / Tourist | 4 | 461 | 1.0991e-4 |
| DNA / Transib | 2 | 262 | 6.2467e-5 |
| DNA / Zator | 1,344 | 107,987 | 2.5747e-2 |
| DNA | 61 | 4,281 | 1.0207e-3 |
| RC / Helitron | 8,703 | 777,310 | 0.18533 |
| ARTEFACT | 1 | 68 | 1.6213e-5 |

| **Transposon families** | **Copy number** | **Total bases** | **Fraction in the genome** |
| --- | --- | --- | --- |
| LINE / CR1 | 6,667 | 799,149 | 0.19054 |
| LINE / CRE | 112 | 3,076 | 7.3339e-4 |
| LINE / Dong-R4 | 28 | 252,483 | 6.0198e-2 |
| LINE / I | 366 | 72,441 | 1.7272e-2 |
| LINE / Jockey | 416 | 85,894 | 2.0479e-2 |
| LINE / L1 | 19 | 1,260 | 3.0041e-4 |
| LINE / L1-Tx1 | 27 | 1,274 | 3.0375e-4 |
| LINE / L2 | 1,978 | 343,991 | 8.2015e-2 |
| LINE / LOA | 116 | 17,314 | 4.1280e-3 |
| LINE / Penelope | 46 | 7,164 | 1.7081e-3 |
| LINE / Proto2 | 80 | 17,363 | 4.1397e-3 |
| LINE / R1 | 1,247 | 357,763 | 8.5299e-2 |
| LINE / R2 | 9 | 520 | 1.2398e-4 |
| LINE / RTE | 1 | 62 | 1.4782e-5 |
| LINE / RTE-BovB | 1,798 | 260,398 | 6.2085e-2 |
| LINE / RTE-RTE | 367 | 146,687 | 3.4973e-2 |
| LINE / RTE-X | 7 | 403 | 9.6084e-5 |
| LTR / Copia | 739 | 206,621 | 4.9263e-2 |
| LTR / DIRS | 101 | 31,866 | 7.5976e-3 |
| LTR / Gypsy | 2,289 | 592,512 | 0.14127 |
| LTR / Pao | 843 | 172,305 | 4.1081e-2 |
| LTR | 124 | 27,459 | 6.5468e-3 |
| SINE | 11,379 | 1,181,196 | 0.28162 |
| SINE / 5S-Deu | 3 | 365 | 8.7024e-5 |
| SINE / tRNA | 21,211 | 3,326,291 | 0.79306 |
| SINE / tRNA-CR1 | 11,874 | 1,664,971 | 0.39697 |
| SINE / tRNA-Deu | 15 | 1,414 | 3.3713e-4 |
| SINE / tRNA-I | 5 | 198 | 4.7208e-5 |
| Unknown | 34 | 3,251 | 7.7511e-4 |
| **Total** | **79,624** | **11,725,628 bp** | **2.7956 %** |
