# Supplementary figures and images for: Somatic piRNAs and Transposons are Differentially Expressed Coincident with Skeletal Muscle Atrophy and Programmed Cell Death
Source: Front Genet. 2021 Dec 22;12:775369. doi: 10.3389/fgene.2021.775369 (PMC8730325; doi:10.3389/fgene.2021.775369)

Figure S1

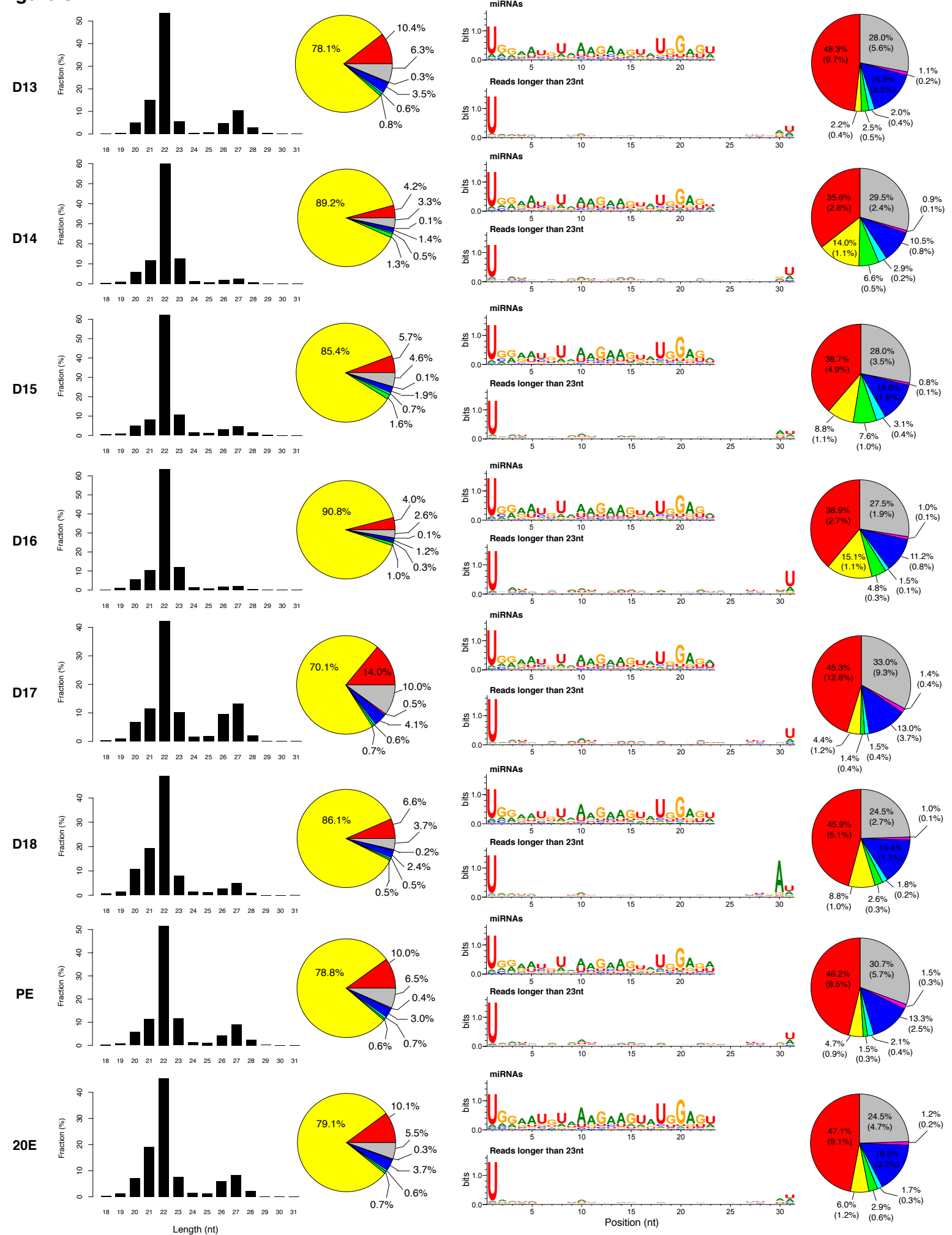

Figure S2

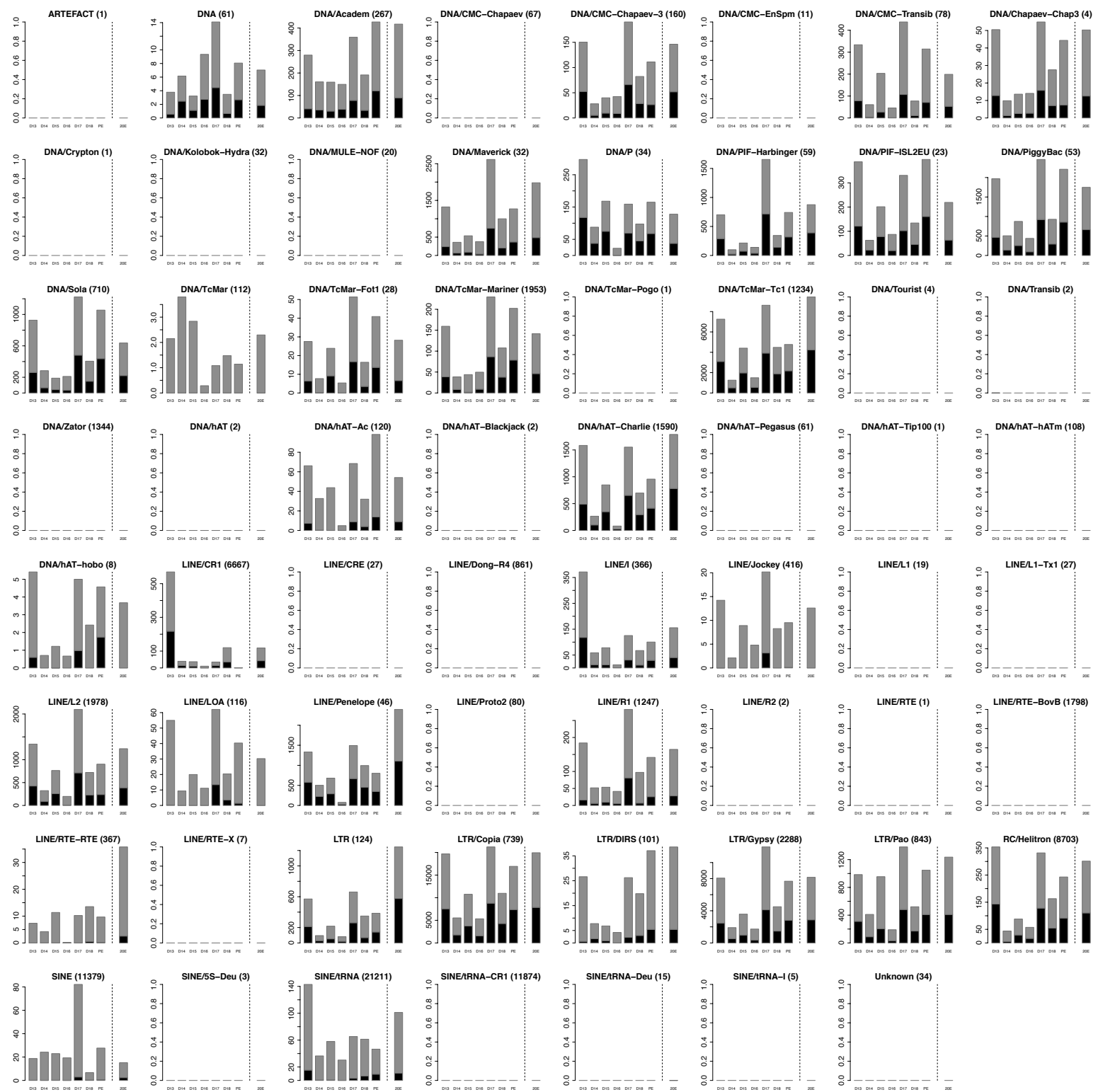

\* Y-axis: piRNA abundance (ppm)

Figure S3

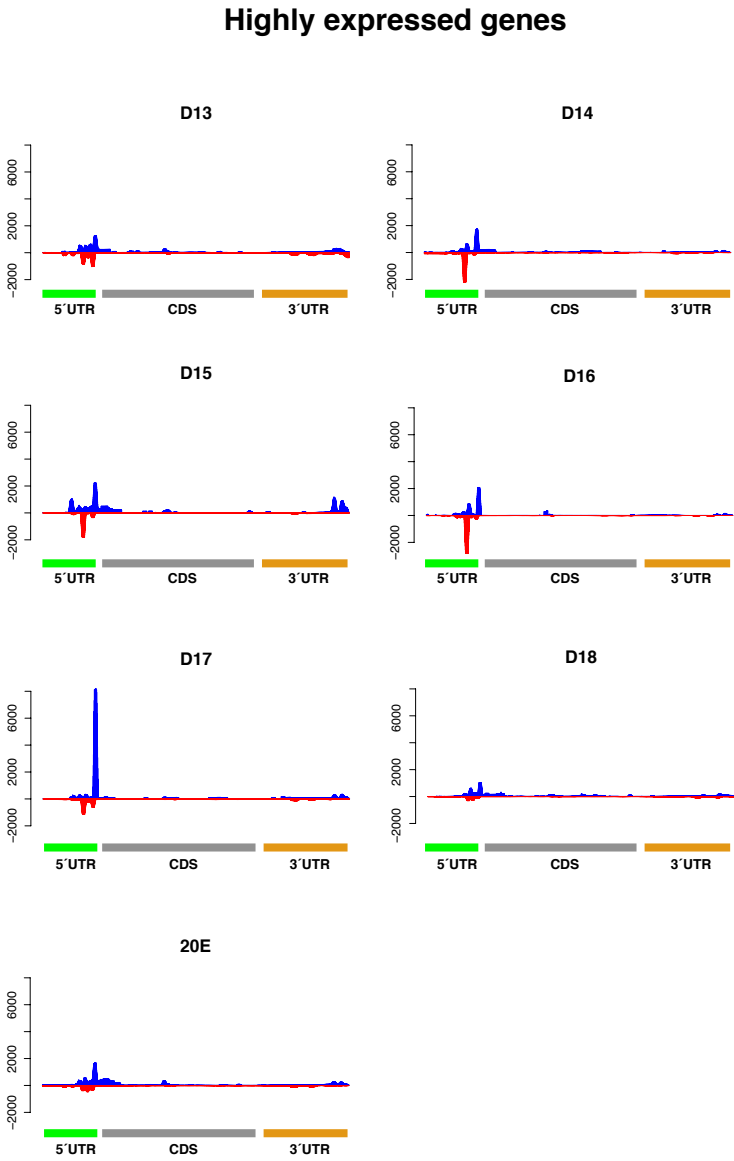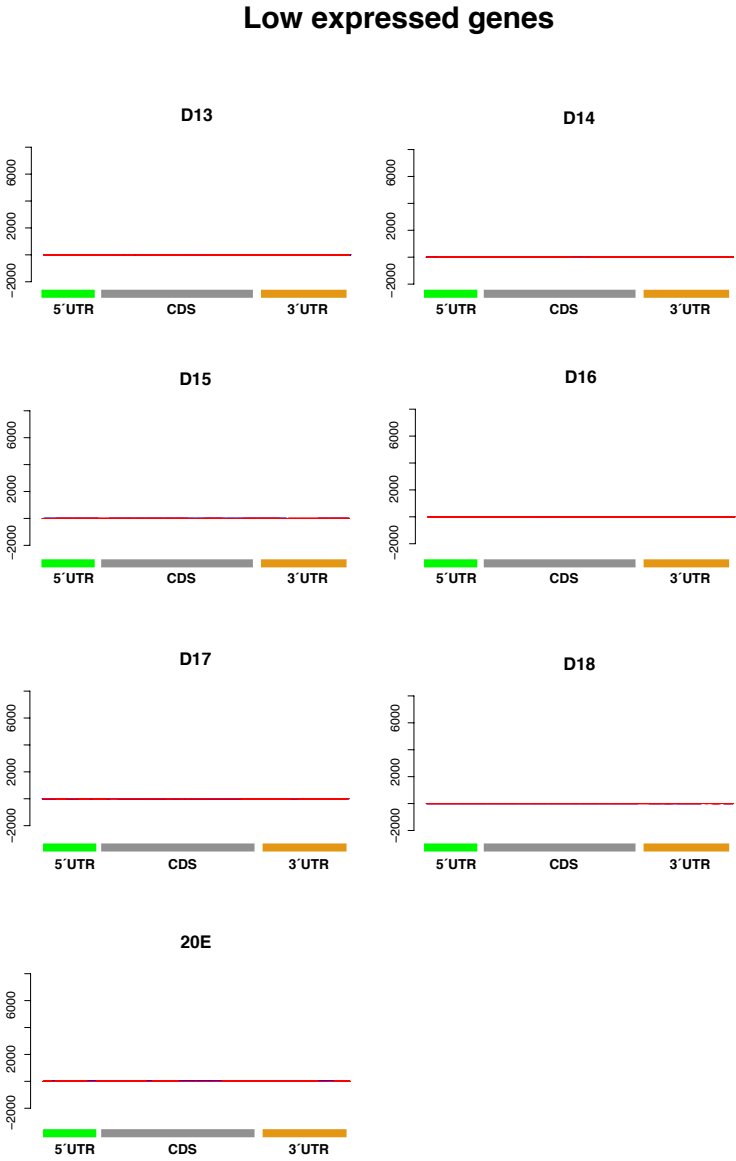

Figure S4

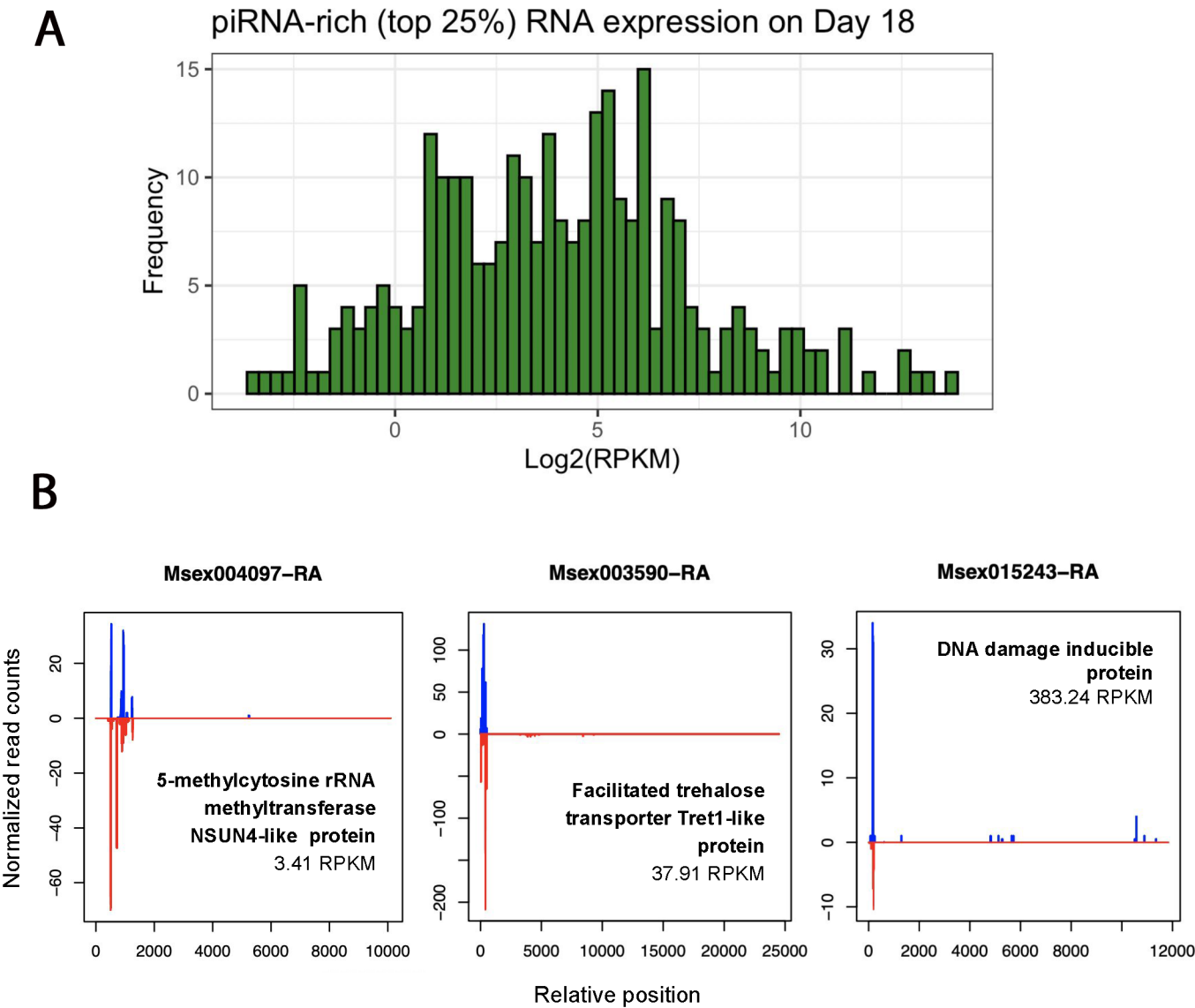

Figure S5

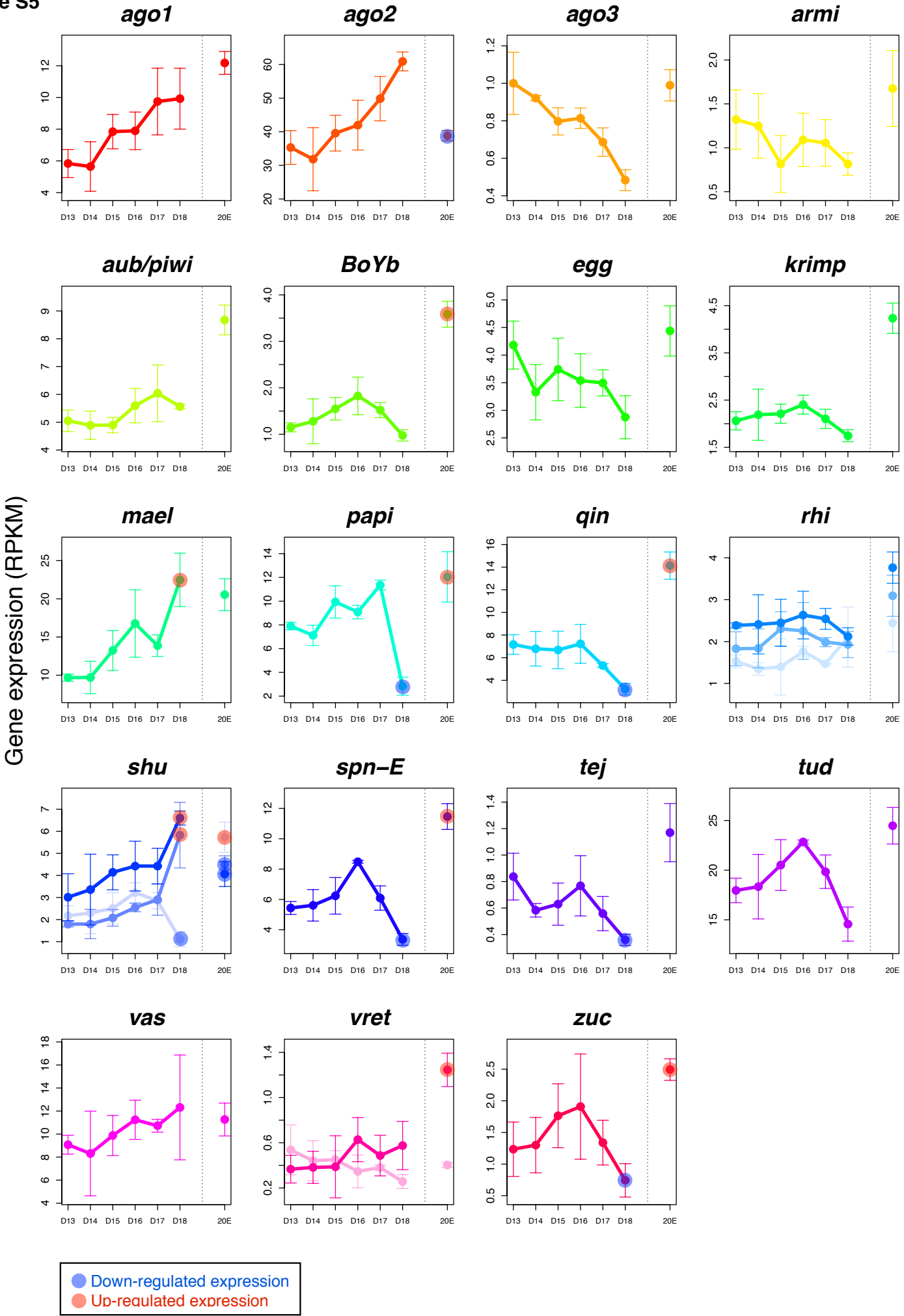

Supplement: Supplementary file 3 [file DataSheet1.PDF]
