# Supplementary material for: Somatic piRNAs and Transposons are Differentially Expressed Coincident with Skeletal Muscle Atrophy and Programmed Cell Death
Source: Front Genet. 2021 Dec 22;12:775369. doi: 10.3389/fgene.2021.775369 (PMC8730325; doi:10.3389/fgene.2021.775369)
Supplement: Supplementary file 4 [file Table3.DOCX]

**Somatic piRNAs and Transposons are Differentially Expressed Coincident With Skeletal Muscle Atrophy and Programmed Cell Death**

Junko Tsuji^1,a^, Travis Thomson^2,3^ , Christine Brown^4^, Subhanita Ghosh^3,b^, William E. Theurkauf^5^, Zhiping Weng^1*^, and Lawrence M. Schwartz ^4*^

^1^Program in Bioinformatics and Integrative Biology, University of Massachusetts Medical School, 368 Plantation Street, Worcester MA 01605, USA

^2^Program in Molecular Medicine, University of Massachusetts Medical School, 364 Plantation Street, Worcester, MA 01605, USA

^3^Department of Neurobiology, University of Massachusetts Medical School, 364 Plantation Street, Worcester, MA 01605, USA

^4^Department of Biology, University of Massachusetts, 611 North Pleasant Street, Amherst MA 01003

^5^Program in Molecular Medicine, University of Massachusetts Medical School, 373 Plantation Street, Worcester MA 01605, USA

^a^Current address: Broad Institute, 415 Main Street, Cambridge, MA 02142

^b^Current address: MRC London Institute of Medical Sciences, Hammersmith Hospital Campus, Du Cane Road, London, W12 0NN, UK

* = co-corresponding authors

**Correspondence to**: Lawrence M. Schwartz

Department of Biology

Morrill Science Center

University of Massachusetts

Amherst, Massachusetts 01003

Phone (413) 545-2435

Fax (413) 545-3243

[LMS@bio.umass.edu](mailto:LMS@bio.umass.edu)

## Supplemental Methods

**Genomic annotations of *Manduca sexta***

We annotate protein-coding genes, transposable elements, low complexity regions, miRNAs and other non-coding RNAs (e.g. rRNA, tRNA, snoRNA, and snRNA) with the following procedures.

- ***Protein coding genes***

We downloaded the nr (non-redundant protein sequence; updated 12/17/2013) and nt (nucleotide sequence; updated 12/16/2013) databases from NCBI website. Using the transcript and protein sequences as queries, we ran BLASTP, BLASTX, and BLASTN with E-value < 10^-4^ (BLAST+ version 2.2.28) against the nr and nt respectively (Altschul et al. 1990). The best BLASTP hits were assigned as gene annotations of *M. sexta*. (Genes that did not identify defined open reading frames were not further analyzed). To add more reliable gene annotations, we also identified the orthologous genes between *M. sexta* and three model organisms: human, mouse, and the fruitfly *Drosophila melanogaster* (*D. melanogaster*). We downloaded proteome datasets for the three organisms from UniProt (release 2013_12) (The UniProt Consortium 2013), and mRNA sequences from CCDS Database (release 15) (Harte et al. 2012) for human and mouse, and from FlyBase (FB2013_06) (Marygold et al. 2013) for *D. melanogaster*. With protein and transcript sequences in *M. sexta*, we ran the three BLAST programs and selected orthologous genes as described the above.

- ***Transposable elements and repetitive regions***

To delineate transposable elements in the genomes of *M. sexta*, we ran RepeatMasker 4.0.3 (Repbase Library: 20130422) (Jurka et al. 2005; Smit et al. 2013) with options of “-species endopterygota” and “-e ncbi” (rmblast 2.2.28). For low-complexity regions we used Tandem Repeat Finder 4.07b. The details of detected transposon families are shown in Table S2.

- ***miRNAs***

We downloaded annotated miRNAs of *M. sexta* in miRBase v20 (Kozomara et al. 2011). We also used candidate miRNAs listed by Zhang X. et al. 2012 (see their Table 5). Those 16 candidate miRNAs are based on the genomic regions that have mapped small RNA reads but low-energy fold-back structure for the miRNA precursors. We also employed miRDeep2 2.0.0.5 with default parameters (Friedländer et al 2008) to predict miRNAs using our unoxidized small RNA-seq datasets.

- ***Other non-coding RNAs***

For the annotations of other non-coding RNAs (e.g. rRNA, tRNA, snoRNA, and snRNA), we mapped all sequences downloaded from Rfam 11.0 (Gardner et al. 2009) to the *M. sexta* genome using LAST (version 318) (Kielbasa et al. 2011) and assigned the best hits (E-value < 10^-4^) to the non-coding RNA annotations in *M. sexta*. In addition, for tRNA and rRNA annotations, we combined the prediction results of tRNAscan-SE (Lowie and Eddy 1997) and the results of RepeatMasker with the Rfam results.

**Primers for constructing small RNA sequencing libraries**

Reverse Barcoded Primers (SmRNAZZPCR**Id5)**: 5’-CAAGCAGAAGACGGCATACGAGAT**CACTGT**GTGACTGGAGTTCCTTGGCACCCGAGAATTCCA-3’

Forward Primer:

5’-AATGATACGGCGACCACCGAGATCTACACGTTCAGAGTTCTACAGTCCGA-3’

5' Adapter: 5’-GUUCAGAGUUCUACAGUCCGACGAUC-3’

3' Adapter: 5’-rAppTGGAATTCTCGGGTGCCAAGG/ddC/-3’
